# Use of Benzodiazepines and Z-Drugs in Multiple Sclerosis

**DOI:** 10.3389/fneur.2022.874724

**Published:** 2022-04-14

**Authors:** Ruth Ann Marrie, John D. Fisk, Randy Walld, James M. Bolton, Jitender Sareen, Scott B. Patten, Alexander Singer, Lisa M. Lix, Carol A. Hitchon, Renée El-Gabalawy, Alan Katz, James J. Marriott, Charles N. Bernstein, the CIHR Team in Defining the Burden Managing the Effects of Psychiatric Comorbidity in Chronic Immunoinflammatory Disease

**Affiliations:** ^1^Department of Internal Medicine, Max Rady College of Medicine, Rady Faculty of Health Sciences, University of Manitoba, Winnipeg, MB, Canada; ^2^Department of Community Health Sciences, Max Rady College of Medicine, Rady Faculty of Health Sciences, University of Manitoba, Winnipeg, MB, Canada; ^3^Nova Scotia Health and the Departments of Psychiatry, Psychology and Neuroscience, and Medicine, Dalhousie University, Halifax, NS, Canada; ^4^Manitoba Centre for Health Policy, Max Rady College of Medicine, Rady Faculty of Health Sciences, University of Manitoba, Winnipeg, MB, Canada; ^5^Department of Psychiatry, Max Rady College of Medicine, Rady Faculty of Health Sciences, University of Manitoba, Winnipeg, MB, Canada; ^6^Department of Community Health Sciences, Cumming School of Medicine, University of Calgary, Calgary, AB, Canada; ^7^Department of Family Medicine, Max Rady College of Medicine, Rady Faculty of Health Sciences, University of Manitoba, Winnipeg, MB, Canada; ^8^Department of Clinical Health Psychology, Max Rady College of Medicine, Rady Faculty of Health Sciences, University of Manitoba, Winnipeg, MB, Canada; ^9^Department of Anesthesiology, Perioperative Medicine and Pain, Max Rady College of Medicine, Rady Faculty of Health Sciences, University of Manitoba, Winnipeg, MB, Canada

**Keywords:** multiple sclerosis, benzodiazepines, Z-drugs, cohort, psychiatric comorbidity

## Abstract

**Objective:**

Use of benzodiazepines and Z-drugs (non-benzodiazepine sedative hypnotics) is controversial due to adverse health outcomes in the general population. However, little is known about their use in people with multiple sclerosis (MS). We estimated the incidence and prevalence of benzodiazepine and Z-drug use (jointly BZD) in the MS population as compared to an age-, sex- and geographically-matched population without MS, and examined the association of mood/anxiety disorders with the use of BZD over a twenty-year period.

**Methods:**

Using administrative data from Manitoba, Canada, we identified 2,985 persons with incident MS and 14,891 persons without MS matched 5:1 on sex, birth year and region. We applied validated case definitions to identify persons with any mood/anxiety disorder. Dispensations of BZD were identified. To assess the association between MS, mood/anxiety disorders and BZD use we constructed generalized linear models adjusting for age, sex, index year, socioeconomic status, urban/rural residence, physical comorbidities, and health care use. We also examined patterns of BZD use.

**Results:**

In 2016, the crude incidence of benzodiazepine use in the MS cohort was 2.10% (95%CI: 1.43–2.98%), 1.49-fold higher than in the non-MS cohort (1.41%; 95%CI: 1.18–1.67%). The crude incidence of Z-drug use in the MS cohort was 1.77% (95%CI: 1.20–2.51%), 1.78-fold higher than in the non-MS cohort (0.99%; 95%CI: 0.81–1.21%). After adjusting for covariates, among individuals without an active mood/anxiety disorder, the MS cohort had a 39% increased incidence rate of benzodiazepine use and a 72% increased incidence rate of Z-drug use as compared to the non-MS cohort. Among individuals with an active mood/anxiety disorder, the incidence of BZD use did not differ between the MS and non-MS cohorts. A higher proportion of people with MS used BZD for ≥6 months than people without MS.

**Conclusion:**

Use of BZD is more common in people with MS than in general population controls, and use of these agents is in persons with MS is often chronic.

## Introduction

Psychiatric comorbidity is common in people with multiple sclerosis (MS). In a Canadian study of people with MS, the prevalence of depression was 20%, while the prevalence of anxiety disorders was 9%, 58% higher than in age, sex and geographically matched controls without MS (1). Sleep disorders are also common in people with MS, with one study reporting that 22% met International Classification of Sleep Disorders criteria for chronic insomnia disorder (2). In the general population, benzodiazepines are widely used to manage anxiety disorders and short-term sleep disorders while the related Z-drugs (e.g., zopiclone) are approved for use in sleep disorders. However, the widespread use of benzodiazepines and Z-drugs is controversial due to the adverse health outcomes with which they have been associated in the general population (3). In the MS population, the extent of use of benzodiazepines and Z-drugs has received relatively little attention. One population-based Swedish study found that people with MS receiving disability pensions were more likely to receive benzodiazepines than controls without MS (4). A Canadian study found that of individuals receiving home care or long-term (nursing home) care, those with MS were more likely to be treated with anxiolytics and sedatives, including benzodiazepines and Z-drugs, than individuals with other neurologic conditions (5).

In 2014, the prevalence of benzodiazepine use in Manitoba, Canada was 6.1%, and this was stable between 1996/97 and 2011/12 (6). While the incidence of benzodiazepine use decreased over time, the incidence of Z-drug use rose (6). Contrasting findings have been observed elsewhere. Based on National Ambulatory Medical Care Survey data in the United States, the percentage of visits for benzodiazepines increased from 3.8 to 7.4% of visits from 2003 to 2015. Increases in benzodiazepine prescribing were observed across all specialties except for psychiatry (7). Benzodiazepines may lead to drug dependence and addiction, and are associated with an increased risk of falls, and with cognitive impairment (8, 9). In elderly individuals, their use is associated with increased motor vehicle accidents and mortality (3, 10). Given the increased propensity for falls and cognitive impairment in the MS population, such adverse effects are a particular concern. This concern is magnified by the aging of the MS population (11). Long-term use of Z-drugs is also not recommended, and they are known to adversely affect driving, and are also associated with falls and cognitive impairment (12).

In September 2020 the standard of practice for prescribing benzodiazepines and Z-drugs in Manitoba was revised due to their major contribution to opioid overdoses and to reduce the harms related to their use (13). Specifically, the standard indicated that prescriptions could be written for a maximum of 3 months, with dispensations for no more than a 1 month supply unless used infrequently or for patients in remote communities who are stable; multiple benzodiazepines and z-drugs, or opioids should not be prescribed together. Patients should be reassessed before prescriptions are refilled and long-term use must be supported by clinical evidence. The standard did not apply to use of these therapies for the management of cancer, palliative care, seizure disorders, psychotic disorders and acute alcohol withdrawal. Earlier implementation of legislation and a monitoring system that regulated narcotics and sedative hypnotics in Ontario, Canada, saw decreased benzodiazepine prescribing (14). A systematic review suggested that prescription monitoring programs were associated with reductions with benzodiazepine prescribing but the effect varied across populations, in some cases disproportionately affecting populations in whom prescribing was appropriate, such as those with seizure disorders, rather than populations in whom prescribing was inappropriate (15).

We aimed to determine the incidence and prevalence of benzodiazepine and Z-drug use in the MS population as compared to an age-, sex- and geographically-matched population without MS, and to examine the association of mood and anxiety disorders with the use of these agents over a twenty year period. In doing so, we sought to provide baseline measures pre-dating practice changes that could guide future studies of the effects of these agents on health outcomes in persons with MS.

## Methods

### Setting

We conducted a retrospective matched cohort study in Manitoba, a Canadian province with a population of ~1.4 million. Health care is universal and publicly funded for medically necessary services. Health service use is recorded in administrative databases.

### Data Sources

The Population Health Data Repository at the Manitoba Centre for Health Policy houses multiple administrative databases, which we accessed for this study. The databases (and data elements used) included the population registry [sex; dates of birth, death and health care coverage; and region of residence (postal code)], physician claims data (service date, and one physician-assigned diagnosis), the discharge abstract database (DAD; hospitalizations, including admission and separation dates, as well as up to 25 diagnoses), and Drug Program Information Network [DPIN; outpatient prescription dispensations, including the date, drug name, and drug identification number (DIN)]. Diagnoses in physician claims are recorded using the International Classification of Diseases (ICD), 9^th^ revision, Clinical Modification (ICD-9-CM). Diagnoses in the DAD are recorded using (ICD)-9-CM codes up to 2004 and ICD 10^th^ revision, Canadian version (ICD-10-CA) codes thereafter. These databases were linked at the individual level using an encrypted unique identifier. We obtained approval to conduct this population-based cohort study from the University of Manitoba Health Research Ethics Board and Manitoba's Health Information Privacy Committee.

### Study Populations

We applied a validated case definition to identify all Manitobans with MS over the period 1984 to 2016. This definition requires an individual to have ≥3 health care encounters for MS (16), as identified based on diagnostic codes for MS in hospitalizations or physician visits or the use of MS-specific disease-modifying therapies. For each person with MS we selected the first demyelinating disease claim and assigned this as the index date. Subsequently we selected a cohort from the general population matched 5:1 on sex, year of birth within ± 5 years, and forward sortation area (first 3 digits of postal code). The matched cohort excluded anyone with any ICD-9-CM and ICD-10-CA diagnosis codes for demyelinating disease or use of any MS-specific disease-modifying therapies, as well as individuals with diagnosis codes for inflammatory bowel disease and rheumatoid arthritis because we were conducting parallel studies for these diseases. Each control was assigned the index date of their matched case. These constituted prevalent cohorts. From these cohorts, we selected incident cases with an index date of 1997 or later. Since DPIN data began in the fiscal year 1995/96 this allowed a 1 year run-in period to determine whether medication use was truly incident (new) after the index date. Benzodiazepines are used for treatment of seizure disorders but their use for this indication was not the focus of our study. Moreover, the practice standard limiting use of benzodiazepines did not apply to the use of these therapies for seizure disorders. Therefore, we also excluded individuals with who had diagnostic codes for seizure disorder (ICD-9 345, ICD-10 G40/G41) before the index date. To preserve the matching balance their matches were also excluded. As well, anyone who developed a seizure disorder following the index date was censored when their first seizure disorder diagnostic code appeared.

### Psychiatric Comorbidity

Consistent with prior work using these cohorts, we applied validated case definitions to identify persons with any diagnosed mood or anxiety disorder, which included ≥1 of depression, anxiety, or bipolar disorders (17). The date of the first claim for each condition was considered the diagnosis date. We assessed psychiatric status annually to account for the relapsing and remitting nature of mood/anxiety disorders (18–20), and potential impacts on pharmacotherapy use. We considered the affected person to be an “active” case if there were ≥2 physician claims or one hospital claim with a diagnosis code for the mood/anxiety disorder in that year; for hospital claims the mood/anxiety disorder was required to be the most responsible diagnosis (21). Prescription claims alone were not considered a marker of “active” disease.

### Outcomes

The DIN captured in DPIN was linked to the World Health Organization's Anatomical Therapeutic Chemical (ATC) Classification System (22) from which we identified benzodiazepines (N05BA12, N05BA08, N05BA02, N05BA09, N03AE01, N05BA01, N05BA17, N05BA06, N05BA04, N05CD07, N05CD05) and Z-drugs (N05CF01, N05CF02, N05CF03, N05CF04) available in Canada. Throughout, we use the abbreviation “BZD” to jointly refer to benzodiazepines and Z-drugs. We defined new (incident) BZD users as those having no use of any BZD for at least 1 year before first dispensation. Prevalent BZD users were defined as individuals with at least one BZD dispensation in the year of interest.

We assessed time from the first BZD dispensation to discontinuation of therapy as defined by a gap of at least 90 days between dispensations (23), as measured using dispensation dates and the number of days supplied. We also described patterns of use (10). To ensure similar follow-up, we completed these analyses in cohort members who had at least 5 years of follow-up after the index date. We aimed to describe short-term use (<6 months) and chronic use (≥6 months) within that 5 year period since a prior systematic review identified 6 months use as the most common definition of chronic (long-term) use (24). We report the percentage [95% confidence interval (95%CI)] for (i) single dispensation; (ii) continuous use for at least 12 weeks; (iii) cumulative use of at least 12 weeks in any 1 year period; (iv) continuous use for at least 6 months; (v) cumulative use of at least 6 months in a 1 year period; and (vi) intermittent use, defined as prescriptions in two or more years, with a gap of at least 1 year between dispensations. Because of the 5-year period, discontinuation of BZD could be followed by re-initiation of therapy, and the total percentages could exceed 100%.

To place our findings in the context, we also report the frequency of use of classes of medications that may be used for spasticity, pain or mood [dantrolene, baclofen, tizanidine, cyclobenzaprine, botulinum toxin, gabapentin, pregabalin, cannabinoids, tricyclic antidepressants, selective serotonin reuptake inhibitors (SSRI) and selective norepinephrine serotonin reuptake inhibitors (NSRI)]. These were identified based on at least one dispensation.

### Covariates

Covariates in the analyses included current age [18–24 (reference group), 25–44, 45–64, ≥65], sex (male as reference group), index year (continuous), region of residence [urban, rural (reference)], disease duration from the index date (continuous), annual number of physician visits (continuous), annual number of classes (types) of prescription medications dispensed (including disease-modifying therapies), at the 4^th^ level of the ATC system (e.g., by chemical subgroup) after excluding BZD (0–1, 2–3, ≥4), and number of physical comorbidities [0 (reference group), 1, ≥2]. We also included socioeconomic status in index year (SES, continuous) which was determined by linking postal code to dissemination-area level census data. From this we calculated the Socioeconomic Factor Index version 2 (SEFI-2) which integrates average household income, high school education rates, unemployment rates and percent of single parent households into a single score, where scores <0 indicate higher SES (25). The number of physician visits and number of classes of medications dispensed were included to account for differences in health care use between cohorts. Physical comorbidity counts were obtained using the John Hopkins Adjusted Clinical Group System Aggregated Diagnosis Groups (ADGs™), which is a case-mix system developed in the United States that also predicts health care use in Canadian populations (26, 27). We used chronic (not time-limited) major physical ADGs, consistent with our prior work (28). For analyses of incident BZD use we assessed physical comorbidity, number of physician visits, and number of medication classes used in the year before the first dispensation. These were updated annually for the prevalent use analyses.

### Analysis

We summarized the characteristics of the MS and matched cohorts using means [standard deviation (SD)], medians [interquartile range (IQR)] and frequency (percent). Annually, we estimated the crude incidence rate (new users) of any BZD over the study period, overall and stratified by sex, and age (18–44, 45–64, ≥65 years). In addition to crude incidence rates, we report incidence rates that are age and sex-standardized to the 2010 Canadian population (from the Statistics Canada census) and 95%CI based on a negative binomial distribution. We compared rates between the cohorts using rate ratios and 95%CI. To assess the association between cohort (MS vs. not MS), mood/anxiety disorders (active vs. inactive/absent) and use of BZD we constructed generalized linear models using a binomial distribution and the log of person-time as the model offset. To account for repeated observations in individuals in the prevalence analyses we used generalized estimating equations with an exchangeable correlation structure. Covariates in these models included age, sex, index year, SEFI2, region, physical comorbidities, and measures of health care use as described above.

Statistical analyses were conducted using SAS V9.4 (SAS Institute Inc., Cary, NC).

## Results

The MS cohort included 2,985 persons and the non-MS cohort included 14,891 persons. Most were female and living in urban centers (Table 1). One-quarter of the MS cohort had their index date in the interval from 2012 to 2017. In the year before the first BZD dispensation, of 792 people with MS, 224 (28.3%) had received at least one prescription dispensation of medications that may be used for spasticity, pain or mood; 83 (10.5%) had a dispensation from at least two of those categories. In contrast, among the 2415 people in the non-MS cohort who received a BZD, 455 (17.9%) had received at least one prescription dispensation from any of those categories and 33 (1.3%) had received prescriptions for at least two of those categories. In both cohorts, the most commonly used medications were tricyclic antidepressants, SSRIs or SNRIs (MS *n* = 200, 25.3%; non-MS *n* = 385, 15.1%).

**Table 1:** Characteristics of incident disease cohorts at the time of diagnosis, and matched cohorts at the matched index date.

**Characteristic**	**MS matches** **(*n* = 14,891)**	**MS** **(*n* = 2,985)**
Female, *n* (%)	10,453 (70.20)	2,094 (70.15)
Age at diagnosis, mean (SD)	40.94 (12.82)	40.97 (12.83)
Duration of follow-up from the index date (years), median (IQR)	9.80 (6.04)	9.58 (6.00)
Urban region of residence, *n* (%)	9,710 (65.21)	1,947 (65.23)
Socioeconomic status	−0.18 (0.82)	−0.21 (0.85)
No. physician visits in year pre-index, mean (SD)	3.92 (4.95)	6.26 (6.91)
Cohort sample sizes (%) for ranges of study index years^a^		
1997–2001	4,181 (28.08)	838 (28.07)
2002–2006	3,682 (24.73)	738 (24.72)
2007–2011	3,401 (22.84)	682 (22.95)
2012–2017	3,627 (24.36)	727 (24.36)

*MS, multiple sclerosis; Socioeconomic status, Socioeconomic Factor Index scores; values less than zero indicate higher socioeconomic status; a- earliest index year was 1997 to allow 1 year lookback period as of first availability of prescription claims data*.

### Incidence

**Figure 1:**
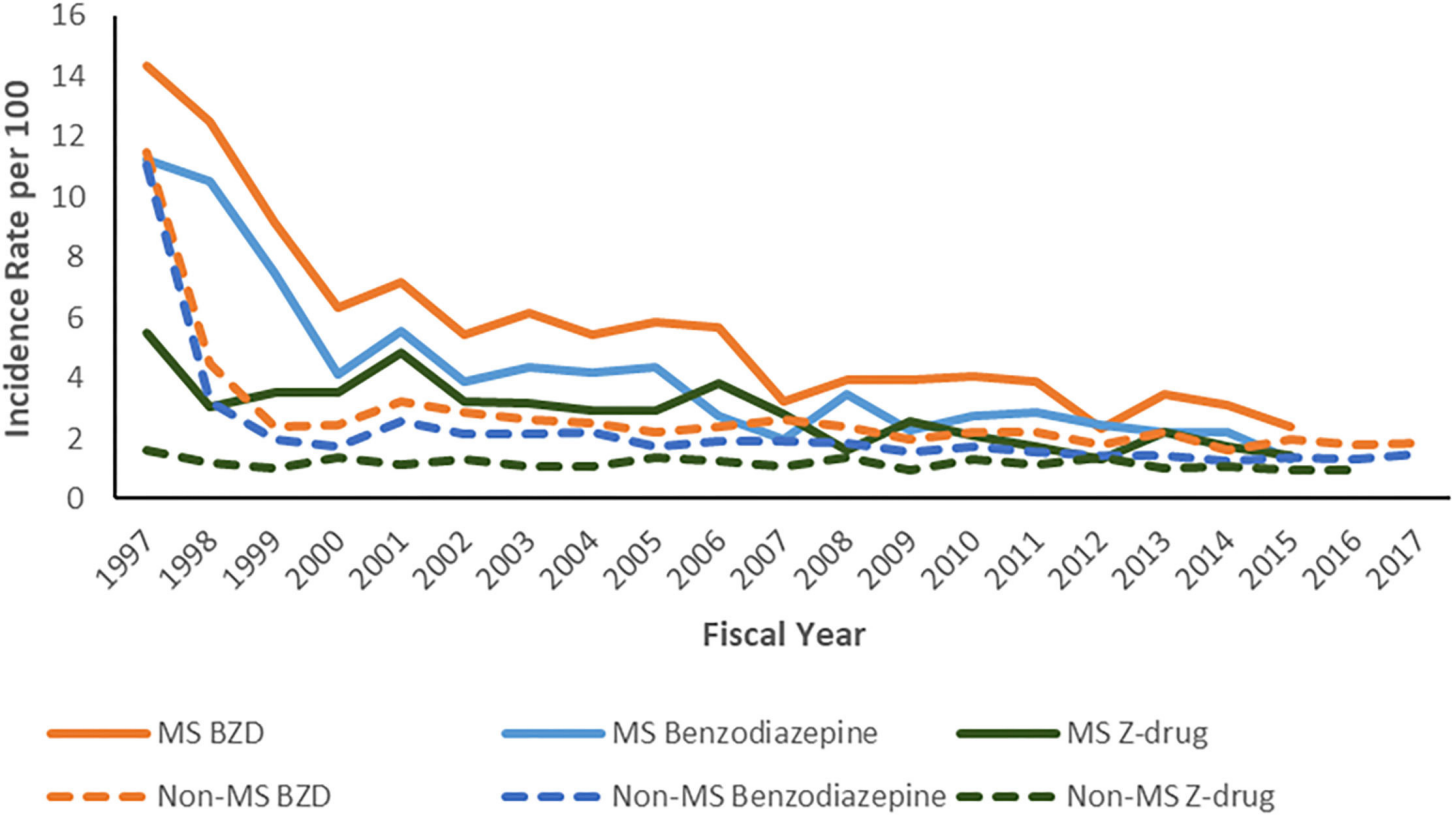
Age- and sex-standardized* annual incidence of benzodiazepine and Z-drug use per 100 persons. *To 2010 Canadian population.

In 2016, the crude incidence of benzodiazepine use in the MS cohort was 2.10% (95%CI: 1.43–2.98%). This was 1.49-fold higher than that of the non-MS cohort (1.41%; 95%CI: 1.18–1.67%). The crude incidence of Z-drug use in the MS cohort was 1.77% (95%CI: 1.20–2.51%). This was 1.78-fold higher than that of the non-MS cohort (0.99%; 95%CI: 0.81–1.21%). After age-standardization, the incidence rate of BZD use declined in the MS cohort over the study period, but it remained persistently elevated as compared to the non-MS cohort (Figure 1).

**Figure 2:**
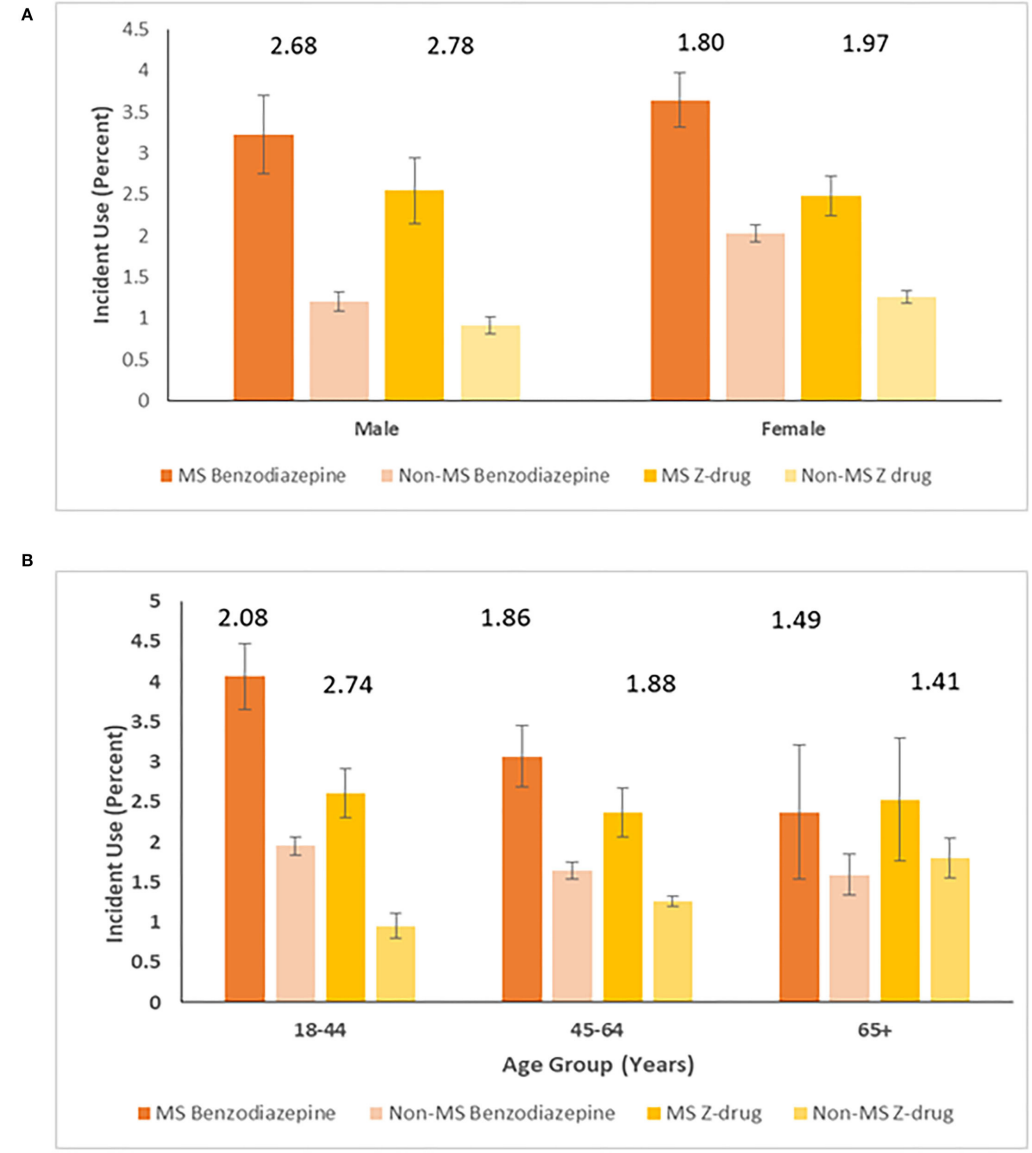
Average annual incidence (95% CI) per 100 person-years of benzodiazepine and Z-drug use stratified by cohort and **(A)** sex* and **(B)** age group*. *Numbers above columns represent ratios comparing MS and non-MS cohorts.

The average annual incidence rate of benzodiazepine use was higher in females than in males in both cohorts (Figure 2A). However, the relative effect of having MS on the incidence of benzodiazepine use in males (RR 2.68; 95%CI: 2.25–3.19) was higher than in females (RR 1.80; 95%CI: 1.62–1.98, *p* < 0.0001). The rate ratio comparing the MS and non-MS cohorts was also highest among those aged 18–44 years (RR 2.08; 95%CI: 1.86–2.34), decreasing among those aged 45–64 years (1.86; 95%CI: 1.62–2.14) and was lowest among those aged 65 years and older (RR 1.49; 95%CI: 1.02–2.19) (Figure 2B). Findings were similar for the Z-drugs.

After stratifying by the presence of a mood/anxiety disorder, the age and sex-standardized incidence of benzodiazepine use over the study period was higher in the MS cohort than in the non-MS cohort although the magnitude of this differential effect was higher among those without a mood/anxiety disorder [MS: 3.02%; 95%CI: 2.74–3.35% vs. non-MS: 1.46%; 95%CI: 1.38–1.54%, prevalence ratio (PR) 2.60; 2.45–2.77] relative to those with a mood/anxiety disorder (MS: 6.10%; 95%CI: 4.95–7.50 vs. non-MS: 5.30%; 95%CI: 4.51–6.23%, PR 1.63; 95%CI: 1.48–1.81). These findings were also similar for Z-drug use.

### Prevalence

**Figure 3:**
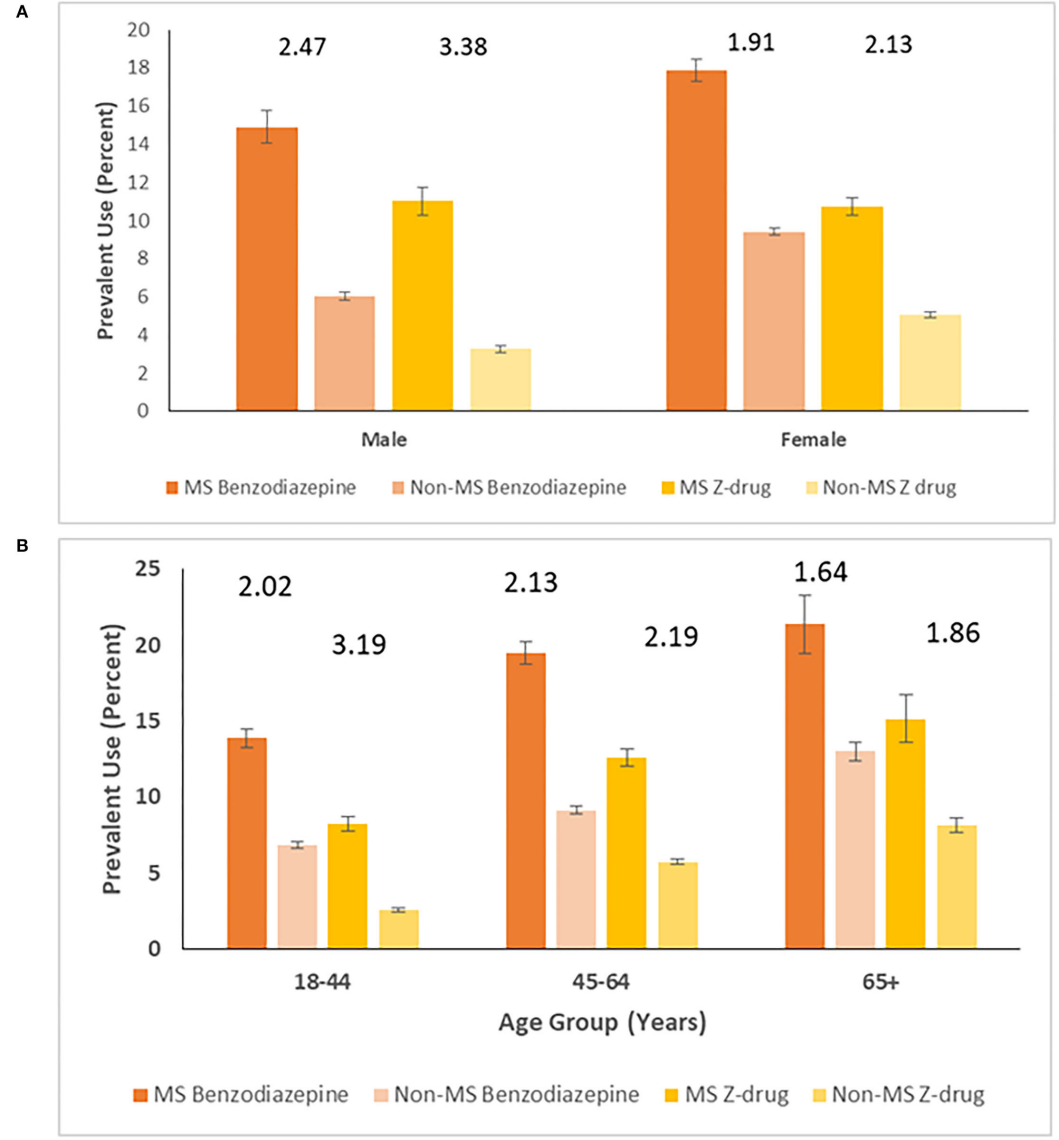
Prevalence (95% CI) of benzodiazepine and Z-drug use per 100 persons in MS and non-MS cohorts, stratified by **(A)** sex* and **(B)** age*. *Numbers above columns represent ratios comparing MS and non-MS cohorts.

Over the study period (1997-2017), the crude prevalence of benzodiazepine use was 17.1% (95%CI: 16.6–17.5%) in the MS cohort and 8.47% (95%CI: 8.32–8.62%) in the non-MS cohort. The crude prevalence of benzodiazepine use was higher in females than in males in both cohorts but the relative effect of having MS on prevalence of benzodiazepine use was higher in males (PR 2.47; 95%CI: 2.31–2.64) than in females (PR 1.91; 95%CI: 1.84–1.97, *p* < 0.0001) (Figure 3A). The prevalence of benzodiazepine use rose with age in both cohorts. While the prevalence of benzodiazepine use was higher in the MS cohort than in the non-MS cohort for all age groups, this effect was smallest for those aged ≥65 years (Figure 3B).

After age and sex-standardization, the prevalence of benzodiazepine use over the study period was 16.5% in the MS cohort and was two-fold higher (PR 2.06; 95%CI: 1.98–2.15) than that in the non-MS cohort (8.03%; 7.86–8.20%). After stratifying by the presence of a mood/anxiety disorder, the prevalence of benzodiazepine use remained higher in the MS cohort than in the non-MS cohort, but the magnitude of this effect was greater among those without a mood/anxiety disorder (PR 2.25; 95%CI: 2.14–2.37) than among those with one (PR 1.07; 95%CI: 1.00–1.15).

Findings for Z-drug use paralleled those for benzodiazepine use over the entire study period (1997-2017). Crude prevalence of Z-drug use was 10.8% (10.4–11.2%) in MS cohort vs. 4.56% (4.45–4.66%) in the non-MS cohort. The crude prevalence of Z-drug use was higher in females than in males in both cohorts and the relative effect of having MS on prevalence of Z-drug use was higher in males (RR 2.47; 2.31–2.64) than females (RR 1.91; 1.84–1.97, *p* < 0.0001) (Figure 3A). While the prevalence of Z-drug use was higher in the MS cohort than in the non-MS cohort in all age groups, this effect was smallest for those aged ≥65 years (Figure 3B).

**Figure 4:**
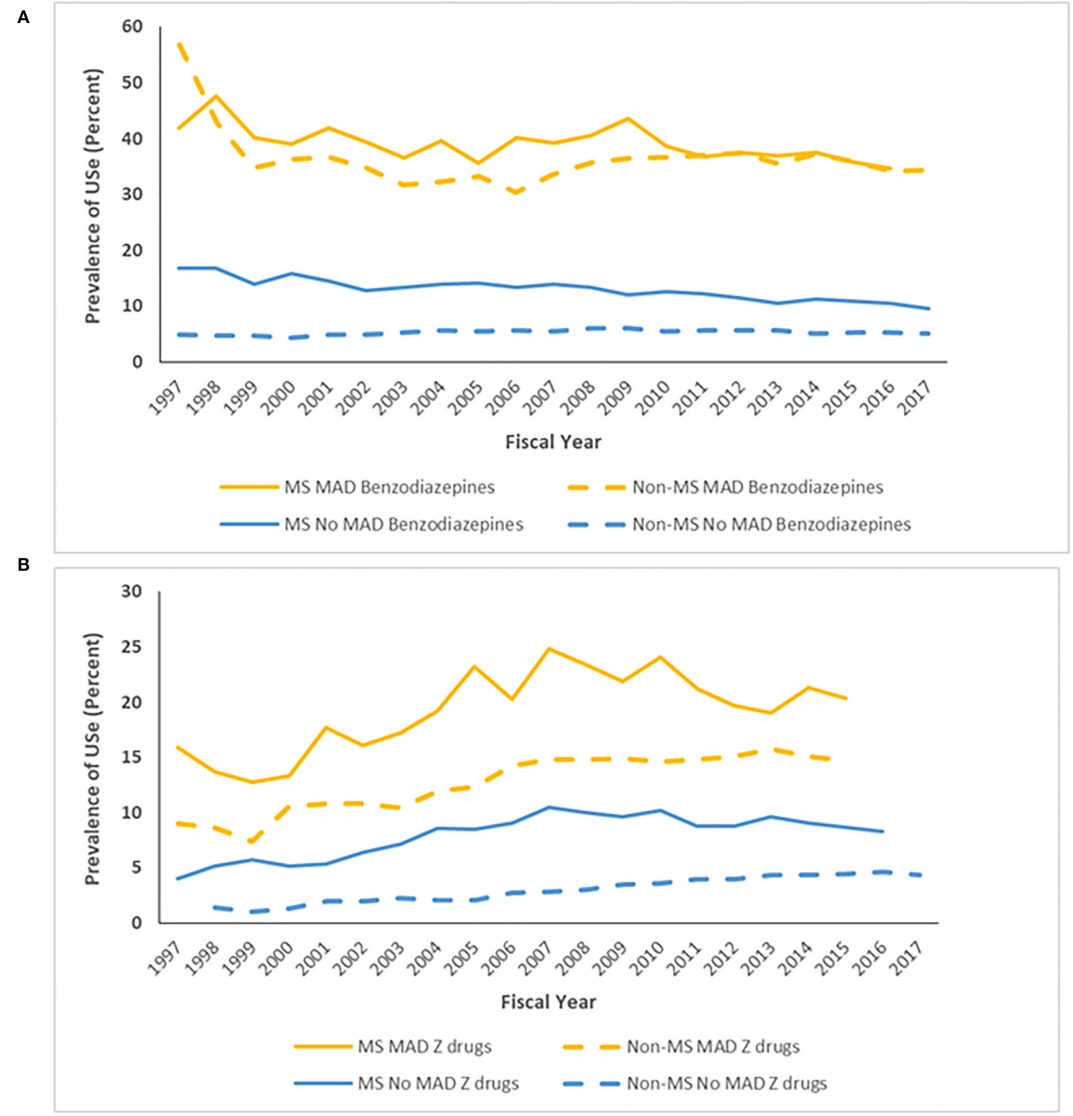
Age- and sex-standardized* prevalence of **(A)** benzodiazepine and **(B)** Z-drug use stratified by cohort and mood/anxiety disorder. *To 2010 Canadian population.

After age and sex-standardization, the prevalence of Z-drug use was 11.0% (95%CI: 10.6–11.5%) in the MS cohort and was over two-fold higher (RR 2.57; 95%CI: 1.98–2.15) than that in the non-MS cohort (4.30%; 95%CI: 4.18–4.42%). After stratifying by the presence of a mood/anxiety disorder, the prevalence of benzodiazepine use remained higher in the MS cohort than in the non-MS cohort, but the magnitude of this effect was higher among those without a mood/anxiety disorder (RR 2.60; 95%CI: 2.45–2.77) than among those with one (RR 1.63; 95%CI: 1.48–1.81) (Figure 4).

### Patterns of Benzodiazepine and Z-Drug Use

Among individuals with MS, the median time to discontinuation of benzodiazepines was 30 days (95%CI: 30–30) but among individuals without MS, it was only 20 days (95%CI: 20–25) (log-rank χ^2^ = 31.6, *p* < 0.0001). The same was true for the median time to discontinuation of Z-drugs, where this was 30 days (95%CI: 30–50) for individuals with MS but only 20 days (95%CI: 30–30) (log-rank χ^2^ = 3.70, *p* = 0.054) for individuals without MS.

**Figure 5:**
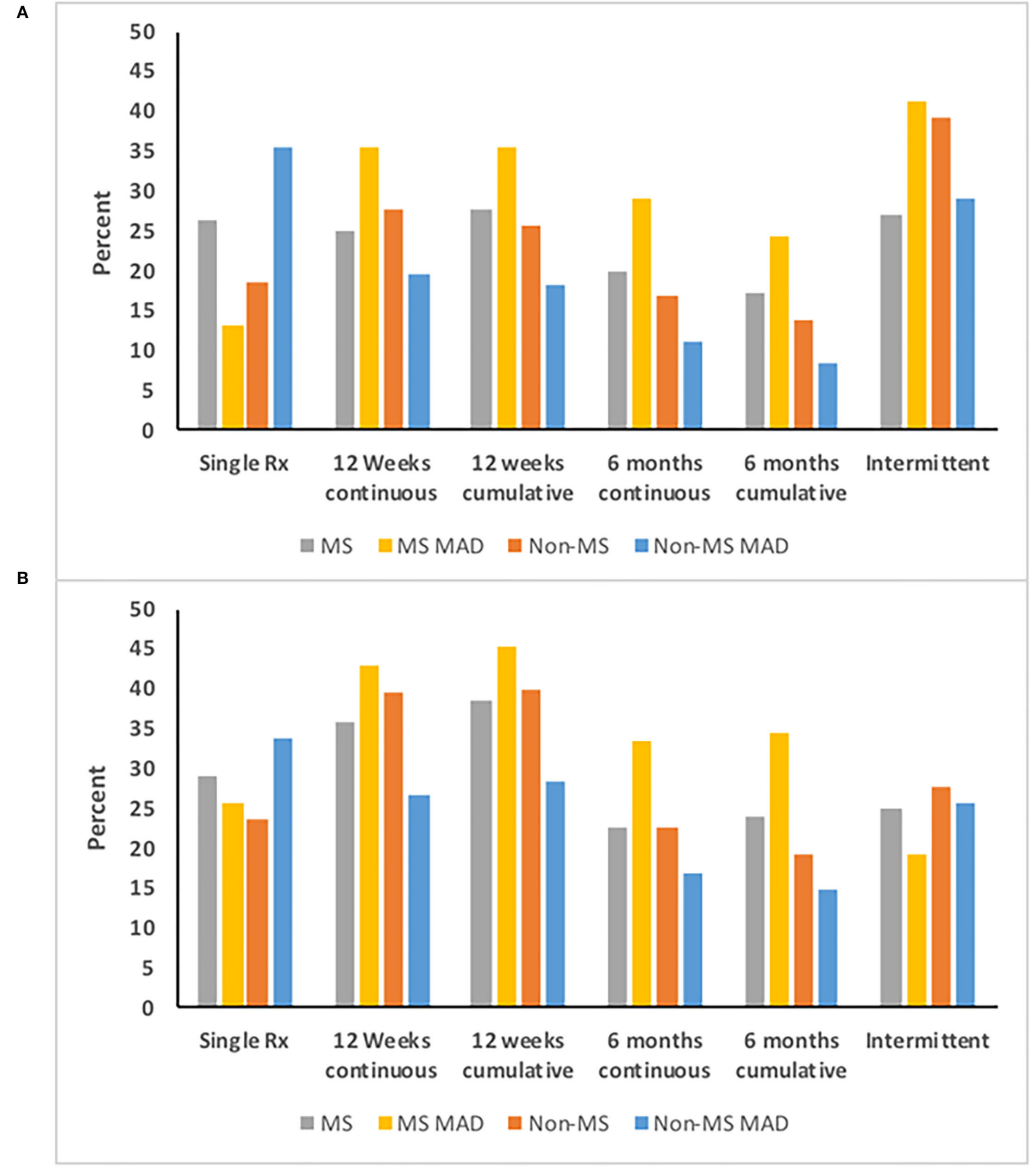
Persistence on **(A)** Benzodiazepines and **(B)** Z-drugs according to multiple sclerosis (MS) and mood/anxiety disorder (MAD) status.

Among incident benzodiazepine users with ≥5 years of follow-up, the patterns of use differed between individuals with and without MS, and between those with and without any mood/anxiety disorder. Among individuals without MS who did not have any mood/anxiety disorder, one-third obtained only a single benzodiazepine dispensation, 11% of individuals used benzodiazepines continuously for 6 months, and one-third used them intermittently. In contrast, one-quarter of individuals with MS who did not have a mood/anxiety disorder used benzodiazepines obtained a single dispensation, one-fifth of individuals used benzodiazepines continuously for 6 months, and 27.1% used them intermittently (Figure 5A).

Among incident Z-drug users with ≥5 years of follow-up, the patterns of use also differed between individuals with and without MS, and with and without any mood/anxiety disorder (Figure 5B). Among individuals without MS who did not have any mood/anxiety disorder, 16.8% of individuals used Z-drugs continuously for at least 6 months. Only one-quarter of individuals used Z-drugs intermittently. Similarly, one-third of individuals only obtained a single Z-drug dispensation. In contrast one-quarter (23.9%) of individuals with MS who did not have a mood/anxiety disorder used Z-drugs for 6 months, as did one-third of individuals with MS and a mood/anxiety disorder.

### Association of Mood/Anxiety Disorder With Use of Benzodiazepines and Z-Drugs

We observed a sub-additive interaction between MS, and a mood/anxiety disorder and use of BZD (Table 2). After adjusting for covariates, among individuals without an active mood/anxiety disorder, the MS cohort had a 39% increased incidence rate of benzodiazepine use and a 72% increased incidence rate of Z-drug use as compared to the non-MS cohort. Among individuals with an active mood/anxiety disorder, the incidence of benzodiazepine use did not differ between the MS and non-MS cohorts. Among individuals with MS, having an active mood/anxiety disorder was associated with an increased incidence of benzodiazepine use, but the magnitude of this association was substantially smaller than that observed for individuals without MS.

**Table 2:** Rate Ratios (95% confidence interval) for the association of multiple sclerosis (MS), active mood/anxiety disorder (MAD) and incidence of benzodiazepine and Z-drug use^*^.

	**Benzodiazepine**	**Z-drugs**	**Combined**
**Incidence**			
MS: Active MAD vs. not	**1.56 (1.29, 1.89)**	**1.40 (1.15, 1.70)**	**1.45 (1.20, 1.75)**
Non-MS: Active MAD vs. not	**2.42 (2.13, 2.74)**	**2.40 (2.11, 2.72)**	**2.46 (2.18, 2.77)**
Active MAD: MS vs. non-MS	0.90 (0.73, 1.11)	1.01 (0.82, 1.23)	0.90 (0.73, 1.10)
No active MAD: MS vs. non-MS	**1.39 (1.24, 1.55)**	**1.72 (1.53, 1.94)**	**1.51 (1.37, 1.67)**
Interaction	**0.65 (0.52, 0.81)**	**0.58 (0.47, 0.73)**	**0.59 (0.48, 0.74)**
**Prevalence**			
MS: Active MAD vs. not	**1.42 (1.31, 1.55)**	**1.17 (1.05, 1.31)**	**1.32 (1.24, 1.40)**
Non-MS: Active MAD vs. not	**2.09 (1.95, 2.24)**	**1.49 (1.36, 1.64)**	**1.82 (1.73, 1.93)**
Active MAD: MS vs. non-MS	**1.27 (1.15, 1.40)**	**1.63 (1.41, 1.89)**	**1.35 (1.25, 1.46)**
No active MAD: MS vs. non-MS	**1.86 (1.71, 2.03)**	**2.07 (1.85, 2.31)**	**1.87 (1.75, 2.01)**
Interaction	**0.68 (0.61, 0.76)**	**0.79 (0.68, 0.91)**	**0.72 (0.67, 0.78)**

* *Adjusted for age (18–44, 45–64, 65+), sex, region, ADG (0, 1, 2+), number of prescription drug classes (0–1, 2–3, 4+), number of physician visits, disease duration, diagnosis year. Bold indicates statistically significant at p < 0.05*.

Overall, the pattern of findings was similar when we examined the association between MS, having a mood/anxiety disorder and prevalent use of BZD (Table 2). The only notable difference was that the prevalence of BZD use was higher in the MS cohort than in the non-MS cohort irrespective of whether there was a comorbid mood/anxiety disorder or not.

## Discussion

In this population-based study using twenty years of administrative data, we estimated the incidence and prevalence of use of BZD among individuals with MS. On average 1 in 6 people with MS used a benzodiazepine annually, and 1 in 10 used a Z-drug annually. One in five people with MS started taking a benzodiazepine annually, slightly more than the number who initiated a Z-drug. We found that use of BZD was higher in people with MS than without MS, overall, in males and females, and in all age groups. In the MS and non-MS cohorts BZD use was also higher among individuals with a mood/anxiety disorder than individuals without a mood/anxiety disorder, even after accounting for age, sex, socioeconomic status, region, physical comorbidities, health care use, disease duration, and index year. Individuals with MS generally used BZD longer than individuals without MS. Over half of those with MS who had an active mood/anxiety disorder used benzodiazepines for at least 6 months, and almost one in ten used Z-drugs for at least 6 months. Thus the higher prevalence of BZD use in MS seems partially related to a higher incidence of BZD use and to a longer duration of use. This suggests that strategies are needed to reduce new exposures to BZDs and to reduce duration of use through deprescribing.

Little prior work has assessed the incidence or prevalence of BZD use in the MS population. To date, one study of 190 individuals attending a single MS clinic in the United States, found that 17.3% of participants reported using a benzodiazepine (29). In a cross-sectional Canadian study, 22.4% adults with MS using home care used anxiolytics and 16.5% used sedatives (5). Use of anxiolytics and sedatives was slightly lower in the long-term care setting than in the home care setting but was higher in those with MS than in adults with other neurologic conditions in both settings. Among Swedish adults granted a disability pension, people with MS had 72% increased odds of receiving a benzodiazepine prescription (4). The incidence of BZD use decreased over time in both cohorts. This is consistent with observations in the general population in other health regions such as Spain and France (30, 31). In a multi-jurisdictional study conducted over the period 2010 to 2016, incident benzodiazpine used decreased from 2.6 to 1.7% among US veterans aged 65 years and older, and from 6.0 to 4.4% in Ontario, Canada, but did not change in Australia (32). The reductions in BZD use that we observed may reflect changes in prescribing behavior due to increased recognition of the harms of therapy as well as clinical guidelines, such as the Choosing Wisely program, which was adopted in Canada in 2014 (33).

Benzodiazepines are effective for some anxiety disorders and insomnia, but guidelines recommend them for only short-term use (generally 1 to 4 weeks, depending on the treatment indication) (34, 35). Thus single dispensations and intermittent use of benzodiazepines would be consistent with practice guidelines while use lasting 6 months or more is considered long-term use (24). Despite these guidelines, in non-MS populations, depression and anxiety disorders are associated with an increased likelihood of either intermittent or chronic use of benzodiazepines (36). Among individuals with anxiety disorders from primary care clinics in the Canadian province of Quebec, 22.6% used benzodiazepines, of whom most (88.4%) used benozdiazepines for over 12 weeks (37). This definition of long-term use encompassed regular and as needed use. We found that long-term use of benzodiazepines was even higher among individuals with MS, in whom over half of those with a mood/anxiety disorder used benzodiazepines for at least 6 months, and one-third of those without a mood/anxiety disorder also used them for at least 6 months. We are not aware of any comparable information in other studies involving MS populations.

In a telephone survey of people living in Alberta, Canada in 2005-2006, the most common reasons for benzodiazepine use were reported as sleep disorders, anxiety, depression and pain (38). Our observation of a subadditive interaction between MS and a mood/anxiety disorder on BZD, indicates that these agents were used for reasons other than, or in addition to mood/anxiety disorders for the MS population. Sleep disturbance, including insomnia, is common in people with MS although predominantly in those with depression or anxiety (39). Restless legs syndrome and nocturnal spasticity are also indications for benzodiazepine use, and often affect people with MS. A multicentre observational study found that 22.3% of people with MS met the diagnostic criteria for chronic insomnia disorder (2). Factors associated with insomnia in that study sample included female sex, physical comorbidities, anxiety and fatigue (2). Although insomnia may be a common reason for using BZD, their effectiveness for people with MS is unclear, as in the general population (40). For example, a placebo-controlled clinical trial of eszopiclone in people with MS increased total sleep time but did not improve daytime fatigue (41). Alternative treatments without the adverse effects of sedating medications include cognitive behavioral therapy, which has some evidence of effectiveness for insomnia in people with MS (42). Limited information also supports the use of melatonin for treatment of insomnia in people with MS (43).

Our findings regarding the associations of age and sex with BZD use are consistent with previous general population-based studies. In Canadian and European populations, older age is associated with greater use of BZD with respect to initiation as well as long-term use (24, 38). We found that the association between MS and BZD use diminished with increasing age, suggesting that comorbidity burden and sleep disturbance in the non-MS cohort “caught up” with the MS cohort at older ages, similar to prior observations showing a narrowing of the gap in incidence of some comorbidities such as ischemic heart disease (44). It is also possible that physicians alter their prescribing behavior for BZD as people with MS age and accure more disability. Females use BZD more often than males in the general population, and are more likely to use these therapies chronically (36, 38, 45). This may reflect the higher prevalence of mood/anxiety disorders in females in the general population and higher rates of reporting sleep difficulty (46–48). The differences observed may also reflect increased health-seeking behavior for physical and mental health concerns by females (48). Given the predominance of females in the MS population, and sex-specific differences in use of BZD, we incorporated a matched non-MS cohort from the general population. This allowed us to test for differences in the magnitude of association between sex and BZD use in the MS and non-MS cohorts. Specifically, the disparity in incidence of BZD between males and females was smaller in the MS cohort than in the non-MS cohort, suggesting that MS had a larger effect on increasing the use of BZD in males than in females. This is consistent with prior observations that the disparity in incidence rates of depression between males and females was lower in an MS population than in a matched non-MS population (1), and in a population-based Canadian survey (49).

Limitations of this study should be considered. Although we applied validated case definitions to identify individuals with mood/anxiety disorders, we could not determine the specific indication for which BZD were prescribed. This information could distinguish between clinically appropriate and inappropriate prescribing of these therapies. Similarly, we lacked information about the clinical characteristics of the MS population, such as disability status, that are not captured by health administrative data and we could not examine whether such characteristics are associated with use of these drugs. Administrative data indicate whether medications are dispensed but not whether they are consumed. We did not examine the specialty of the prescriber which may be relevant to future interventions aimed at reducing prescribing of BZD, and to designing appropriate communication and collaborative care models. We also did not compare the doses of BZD used in the MS and non-MS cohorts because of challenges related to defining equivalent doses across differing BZD and differing indications but this may be a useful focus for future work. This study was not designed to examine the adverse consequences of these therapies in the MS population although such consequences are clinically relevant and should be examined in future studies.

## Conclusion

Having a mood/anxiety disorder, being female, and older age are all factors associated with increased use of BZDs, in general. Nonetheless, use of BZD is more common in people with MS than controls from the general population matched for age, sex and location of residence, and use of these agents is in persons with MS is frequently chronic. Use of BZDs can be associated with adverse consequences that are of particular concern for the MS population in which as many as 40–70% of individuals experience cognitive impairment (50), and 50% experience falls (51). Strategies to reduce use of BZDs and offer alternative management strategies for mood/anxiety disorders, sleep disorders and other symptomatic concerns are needed.

## Data Availability Statement

The data analyzed in this study is subject to the following licenses/restrictions: Data used in this article were derived from administrative health data as a secondary use. The data were provided under specific data sharing agreements only approved for use at the Manitoba Centre for Health Policy. The original source data are not owned by the researchers or the Manitoba Centre for Health Policy and as such cannot be provided to a public repository. Where necessary, source data specific to this article or project may be reviewed at MCHP with the consent of the original data providers along with the required privacy and ethical review bodies. Requests to access these datasets should be directed to Charles Burchill, charles_burchill@cpe.umanitoba.ca.

## Ethics Statement

The studies involving human participants were reviewed and approved by University of Manitoba Health Research Ethics Board. Written informed consent for participation was not required for this study in accordance with the requirements of the Health Information Privacy Committee.

## Author Contributions

RM, JF, JB, JS, SP, AS, and CB conceived of the idea. RW conducted the statistical analyses. RM drafted the manuscript. RM, JF, RW, JB, JS, SP, AS, LL, CH, RE-G, AK, JM, and CB revised the manuscript. All authors contributed to the article and approved the submitted version.

## Funding

This study was funded by the Canadian Institutes of Health Research (THC-135234), Crohn's and Colitis Canada and the Waugh Family Chair in Multiple Sclerosis (to RM). CB was supported in part by the Bingham Chair in Gastroenterology. JS was supported by CIHR #333252. AK was supported by a Research Manitoba Chair. LL was supported by a Tier 1 Canada Research Chair. RE-G was supported by University of Manitoba Start-Up Funding. SP holds the Cuthbertson and Fischer Chair in Pediatric Mental Health at the University of Calgary. The sponsors had no role in the design and conduct of the study; collection, management, analysis, and interpretation of the data; and preparation, review, or approval of the manuscript.

## Conflict of Interest

RM receives research funding from: CIHR, Research Manitoba, Multiple Sclerosis Society of Canada, Multiple Sclerosis Scientific Foundation, Crohn's and Colitis Canada, National Multiple Sclerosis Society, CMSC. She is supported by the Waugh Family Chair in Multiple Sclerosis and is a co-investigator on a study funded in part by Roche and Biogen Idec. JB receives research funding from CIHR, Brain and Behavior Research Foundation and the MS Society of Canada. JS receives research funding from CIHR and holds stocks in Johnson and Johnson. SP receives research funding from CIHR, the MS Society of Canada, Roche, Biogen and the Government of Alberta. AS has received financial and in-kind support from an IBM/CIMVHR Advanced Analytics Grant and Calian Inc. LL receives research funds from CIHR and the Arthritis Society. CH has research funds for unrelated studies from UCB Canada and Pfizer. RE-G receives research funds from CIHR, University of Manitoba Start-Up Funds. AK receives research funds from CIHR, the Heart and Stroke Foundation and Research Manitoba. JF receives research funds from CIHR, the MS Society of Canada, Crohn's and Colitis Canada, Research Nova Scotia; consultation and distribution royalties from MAPI Research Trust. JM has conducted clinical trials for Biogen Idec and Roche, and receives research funding from the MS Society of Canada, the MS Scientific Foundation and Research Manitoba. CB has consulted to Abbvie Canada, Amgen Canada, Bristol Myers Squibb Canada, JAMP Pharmaceuticals, Janssen Canada, Pfizer Canada, Roche Canada, Sandoz Canada, Takeda Canada, and has received unrestricted educational grants from Abbvie Canada, Janssen Canada, Pfizer Canada and Takeda Canada. He has been on speaker's bureaus of Abbvie Canada, Janssen Canada, Pfizer Canada and Takeda Canada. The remaining author declares that the research was conducted in the absence of any commercial or financial relationships that could be construed as a potential conflict of interest.

## Publisher's Note

All claims expressed in this article are solely those of the authors and do not necessarily represent those of their affiliated organizations, or those of the publisher, the editors and the reviewers. Any product that may be evaluated in this article, or claim that may be made by its manufacturer, is not guaranteed or endorsed by the publisher.
